# Analyzing 74,248 Samples Confirms the Association Between CLU rs11136000 Polymorphism and Alzheimer’s Disease in Caucasian But Not Chinese population

**DOI:** 10.1038/s41598-018-29450-2

**Published:** 2018-07-23

**Authors:** Zhijie Han, Jiaojiao Qu, Jiehong Zhao, Xiao Zou

**Affiliations:** 1 Innovative Drug Research and Bioinformatics Group, School of Pharmaceutical Sciences, Chongqing University, Chongqing, 401331 China; 2 Institute of Fungus Resources, College of Life Sciences, Guizhou University, Guiyang, 550025 China; 3 College of Pharmacy, Guiyang University of Chinese Medicine, Guian new area, 550025 China

## Abstract

Clusterin (CLU) is considered one of the most important roles for pathogenesis of Alzheimer’s Disease (AD). The early genome-wide association studies (GWAS) identified the CLU rs11136000 polymorphism is significantly associated with AD in Caucasian. However, the subsequent studies are unable to replicate these findings in different populations. Although two independent meta-analyses show evidence to support significant association in Asian and Caucasian populations by integrating the data from 18 and 25 related GWAS studies, respectively, many of the following 18 studies also reported the inconsistent results. Moreover, there are six missed and a misclassified GWAS studies in the two meta-analyses. Therefore, we suspected that the small-scale and incompletion or heterogeneity of the samples maybe lead to different results of these studies. In this study, large-scale samples from 50 related GWAS studies (28,464 AD cases and 45,784 controls) were selected afresh from seven authoritative sources to reevaluate the effect of rs11136000 polymorphism to AD risk. Similarly, we identified that the minor allele variant of rs11136000 significantly decrease AD risk in Caucasian ethnicity using the allele, dominant and recessive model. Different from the results of the previous studies, however, the results showed a negligible or no association in Asian and Chinese populations. Collectively, our analysis suggests that, for Asian and Chinese populations, the variant of rs11136000 may be irrelevant to AD risk. We believe that these findings can help to improve the understanding of the AD’s pathogenesis.

## Introduction

Alzheimer’s Disease (AD) is a commonest kind of neurodegenerative disorders with a complex pathogenesis, and has become one of the leading causes of death in elderly people^1,2^. It is characterized by accumulation and toxic effect of the amyloid β-peptide (Aβ) deposits and neurofibrillary tangles in brain^3^. Previous studies predict that the newly diagnosed AD patients are expected to reach as many as 135 million by 2050 from about 35 million in 2009 around the world if lack of the effective preventive measures^4,5^.

Clusterin (CLU) is considered one of the most important roles for pathogenesis of AD by influencing the structure and neurotoxic effects of Aβ deposits^6–8^, and some of the variants at CLU can affect its expression level in brain^9,10^. Two early genome-wide association studies (GWAS) identified a single nucleotide polymorphism (SNP) rs11136000 (T < C) significantly associated with AD in the CLU gene by analyzing the large-scale Caucasian populations^11,12^. In particular, Harold *et al*.^11^ and Lambert *et al*.^12^ analyzed 11,756 and 14,490 individuals from USA, UK, Ireland, Germany, France, Italy, Spain, Belgium and Finland, respectively, and both of them found that the minor allele variant of rs11136000 can reduce the risk of AD (95% confidence interval (*CI*) of odds ratio (*OR*) less than the value 1).

However, the subsequent studies report consistent^13–18^ and inconsistent^19–28^ results involved in Caucasian, Asian and African populations. For example, by analyzing 268 AD cases and 389 controls from China, Lin *et al*. find that the participants carrying 2 copies of minor allele in rs11136000 are associated with a decreased risk of AD^17^. The consistent result in North American Caucasian population is also identified by Carrasquillo *et al*.^18^. While in Canadian and Korean populations, the rs11136000 is found not associated with AD according to the studies of Bettens *et al*.^24^ and Chung *et al*.^27^, respectively. Then, two independent meta-analysis studies re-assess the results of these GWAS studies published before June 20, 2013 (18 studies) and August 31, 2014 (25 studies), respectively, and both of them found this SNP is significantly associated with AD in populations of Asian and Caucasian^29,30^. But among the subsequent 18 GWAS studies published after August 31, 2014, many of them report inconsistent results in the corresponding populations^31–47^. Moreover, by comparing the selected GWAS articles published before June 20, 2013 in the two meta-analysis studies, we find the selection is incomplete for both of them. In particular, Liu *et al*.^29^ miss two GWAS articles about Caucasian populations^16,24^, and Du *et al*.^30^ miss a GWAS article about Asian population^27^. In fact, through our further investigation, a total five related GWAS articles published before August 31, 2014 are not collected in the two meta-analysis studies^48–52^. In addition, a GWAS study about American and German populations is misclassified to the Asian ethnicity subgroup in Du *et al*.’s study^22^.

We suspected that the small-scale and incompletion or heterogeneity of the samples maybe lead to different results of these studies. In this study, we selected 50 related GWAS studies with large-scale samples from 40 articles (28,464 cases and 45,784 controls, about 40.3% increase over the total number of the previous two meta-analysis studies^29,30^) by searching the PubMed, ClinicalKey, AlzGene, Google Scholar, CNKI, Wanfang and VIP databases, and reevaluated the association between AD and rs11136000 polymorphism in Caucasian, Asian and Chinese population using the method of meta-analysis as previously described^53–63^. The use of more complete and larger scale samples would make the results more reliable.

## Methods and Materials

### Selection of literatures and GWAS studies

All of the possible studies were selected by searching the databases of PubMed (http://www.ncbi.nlm.nih.gov/pubmed, ClinicalKey (https://www.clinicalkey.com/), Wanfang (http://www.wanfangdata.com.cn/), CNKI (http://www.cnki.net/) and VIP (http://www.cqvip.com/) using the keywords: “Alzheimer’s disease”, “rs11136000”, “Clusterin” or “CLU”. The CNKI, Wanfang and VIP are very authoritative and reliable Chinese database. And then, we consulted the related studies collected in AlzGene database (http://www.alzgene.org/) which was a publicly available resource providing the information of AD genetic variants from 1,395 GWAS studies (updated April 18, 2011)^64^. In addition, we further queried references of these identified GWAS studies in previous steps and the articles citing them using the Google Scholar (http://scholar.google.com/).

After that, the appropriate studies were identified by the following criteria: (1) The study is a GWAS to analysis the association of rs11136000 polymorphism and AD. (2) It is a case-control design study. (3) The study provides both of the numbers of cases and controls. (4) The study provides the information about the ethnicity of each individual. (5) The detailed data for rs11136000 genotypes are available in the study.

### Extraction of the related data

We extracted the related data for subsequent analysis from these identified studies: (1) each study’s publication date. (2) The first the author’s name in each of these studies. (3) The numbers of AD patients and controls of each study. (4) The sample’s ethnicity of each study. (5) The detailed genotype data of rs11136000 polymorphism both in AD patients and controls. (6) The types of genotyping platforms. (7) The key results of each study (i.e. the *OR* value and its 95% *CI*, as well as the corresponding *P* value). Moreover, if these results are not provided in the study directly, we would calculate them by the genotype data using the R program (http://www.r-project.org/).

### Genetic model choice

The rs11136000 polymorphism contains two types of variants (T and C). T is the minor allele and C is the major allele. We assumed that they are the lower and high risk factor for AD, respectively. Then, the dominant model (TT + TC allele versus CC allele), allele model (T versus C) and recessive model (TT versus TC + CC) were used in this study. According to Table 1, all these studies were meta-analyzed using allele model, while only the studies offering CC, CT and TT genotypes data were analyzed using dominant or recessive model.

**Table 1:** Main information of the studies included in this meta-analysis.

Study	Year	Country or institution	Ethnicity	No. of cases	No. of controls	Genotyping platform	Kind of genotype
Jia *et al*.^35^	2017	China	Asian	1,201	4,889	SNaPshot	C/T
Shankarappa *et al*.^34^	2017	India	Asian	243	164	TaqMan	CC/CT/TT
Huang *et al*.^37^	2016	China	Asian	39	56	Sequenom	C/T
Luo *et al*.^41^	2016	China	Asian	109	120	PCR	CC/CT/TT
Rezazadeh *et al*.^39^	2016	Iran	Asian	160	163	PCR	CC/CT/TT
Wang *et al*.^40^	2016	China	Asian	748	760	SNaPshot	CC/CT/TT
Jiao *et al*.^44^	2015	China	Asian	229	318	PCR	CC/CT/TT
Xiao *et al*. (stage 1)^47^	2015	China	Asian	232	373	Sequenom	C/T
Xiao *et al*. (stage 2)^47^	2015	China	Asian	227	378	Sequenom	C/T
Lu *et al*.^28^	2014	China	Asian	493	583	PCR	CC/CT/TT
Chen *et al*.^25^	2012	China	Asian	451	338	Sequenom	CC/CT/TT
Chung *et al*.^27^	2012	Korea	Asian	290	544	TaqMan	C/T
Lin *et al*.^17^	2012	China	Asian	268	389	—	CC/CT/TT
Ma *et al*.^23^	2012	China	Asian	127	143	PCR	CC/CT/TT
Ohara *et al*.^26^	2012	Japan	Asian	824	2,933	Invader assay	CC/CT/TT
Yu *et al*.^21^	2010	China	Asian	324	388	MALDI-TOF mass spectrometry	CC/CT/TT
Seripa *et al*.^33^	2017	Italy	Caucasian	520	569	PCR	CC/CT/TT
Alaylioglu *et al*.^36^	2016	Turkey	Caucasian	183	154	PCR	CC/CT/TT
Montanola *et al*.^38^	2016	Spain	Caucasian	73	88	SNPlex	C/T
Ferrari *et al*.^43^	2015	Italy	Caucasian	37	28	PCR	C/T
Sen *et al*.^45^	2015	Turkey	Caucasian	112	106	TaqMan	CC/CT/TT
Sleegers *et al*.^46^	2015	Belgium	Caucasian	1,295	1,090	PCR	CC/CT/TT
Carrasquillo *et al*.^18^	2014	USA	Caucasian	54	2,424	TaqMan	CC/CT/TT
Pedraza *et al*.^51^	2014	MCADRC	Caucasian	411	2,145	TaqMan	C/T
Roussotte *et al*.^52^	2014	ADNI	Caucasian	173	205	Illumina 610	CC/CT/TT
Mullan *et al*.^49^	2013	Ireland	Caucasian	154	142	TaqMan	C/T
Nizamutdinov *et al*.^50^	2013	Russia	Caucasian	166	128	ABI prism BigDye Terminator	C/T
Bettens *et al*.^24^	2012	Belgium	Caucasian	954	810	PCR	C/T
Bettens *et al*.^24^	2012	France	Caucasian	1,291	608	PCR	C/T
Bettens *et al*.^24^	2012	Canada	Caucasian	304	239	PCR	C/T
Kamboh *et al*.^16^	2012	USA	Caucasian	1,344	1,350	Taqman	CC/CT/TT
Carrasquillo *et al*.^13^	2010	USA	Caucasian	1,819	2,565	Taqman	CC/CT/TT
Corneveaux *et al*.^48^	2010	NIA, MBB	Caucasian	1,019	591	Affymetrix 6.0	C/T
Golenkina *et al*.^20^	2010	Russia	Caucasian	534	702	PCR	CC/CT/TT
Seshadri *et al*.^14^	2010	Spain	Caucasian	1,140	1,209	Illumina 550,370,300 and Affymetrix 500 K	CC/CT/TT
Giedraitis *et al*.^19^	2009	Sweden	Caucasian	79	365	Illumina GoldenGate	CC/CT/TT
Harold *et al*.^11^	2009	USA	Caucasian	1,153	2,187	Illumina 610, 550 and 300	CC/CT/TT
Harold *et al*.^11^	2009	UK,Ireland	Caucasian	2,220	4,833	Illumina 610	CC/CT/TT
Harold *et al*.^11^	2009	Germany	Caucasian	539	824	Illumina 610 and 550	CC/CT/TT
Lambert *et al*.^12^	2009	France	Caucasian	2,039	5,378	Illumina 610	CC/CT/TT
Lambert *et al*.^12^	2009	Italy	Caucasian	1,480	1,263	Taqman and Sequenom	CC/CT/TT
Lambert *et al*.^12^	2009	Spain	Caucasian	748	810	Taqman and Sequenom	CC/CT/TT
Lambert *et al*.^12^	2009	Belgium	Caucasian	1,035	491	Taqman and Sequenom	CC/CT/TT
Lambert *et al*.^12^	2009	Finland	Caucasian	596	650	Taqman and Sequenom	CC/CT/TT
Pedraza *et al*.^51^	2014	MCADRC	African	44	223	TaqMan	C/T
Belcavello *et al*.^42^	2015	Brazil	American	81	161	PCR	CC/CT/TT
Moreno *et al*.^31^	2017	Colombia	Mixed population (Caucasian, African and American)	280	357	PCR	C/T
Santos-Reboucas *et al*.^32^	2017	Brazil	Mixed population (Caucasian, African and mulatto)	174	175	TaqMan	CC/CT/TT
Ferrari *et al*.^15^	2012	UK	Mixed population (Caucasian and African)	342	277	TaqMan	C/T
Gu *et al*.^22^	2011	Indiana	Mixed population (Caucasian and American)	106	98	PCR	CC/CT/TT
All				28,464	45,784		

“CC/CT/TT” means the study offer the data of genotypes CC, CT and TT both in cases and controls. “C/T” means only the data of genotypes C and T are offered in the study. MCADRC: Mayo Clinic Alzheimer’s Disease Research Center; ADNI: Alzheimer’s Disease Neuroimaging Initiative; NIA: National Institute on Aging; MBB: Miami Brain Bank.

### Hardy–Weinberg equilibrium (HWE) test

The HWE test of the rs11136000 polymorphism in AD patient and control groups was performed using a non-continuity correction chi-squared method with the significance level *P* < 0.01 as previously described^65^. Briefly, for the SNP in each case and control group, the simulated *P* values were calculated to measure the deviation from HWE based on 10,000 iterations. The R package ‘Genetics’ was used to perform the HWE test (https://cran.r-project.org/web/packages/genetics/index.html).

### Heterogeneity test

In this study, the heterogeneity among the kinds of populations was measured by the two parameters, *I*^2^ value and Cochran’s *Q*. *I*^2^ value range from 0 to 100%, and it is calculated by Cochran’s *Q* according to the formula ${I}^{2}=\frac{Q-(k-1)}{Q}\times 100 \%$. The Cochran’s *Q* is based on a chi-squared distribution with *k* − 1 degrees of freedom, and *k* means the number of studies. Usually, the extreme, high, moderate and low heterogeneity was considered corresponding to the *I* ^2^ value of >75%, 50–75%, 25–50%, and <25%, respectively. In this study, the threshold of significant heterogeneity was set as *I*^2^ > 50% and *P* < 0.01 according to previous studies^53–56^.

### Meta-analysis in entirety and subgroup

According to the results of heterogeneity test, the random and the fixed effect model were performed when the heterogeneity was significant or not, respectively^66^. We used the R package ‘meta’ to perform the meta-analysis, and determine the significance level of association between rs11136000 and AD through the pooled *OR* value and its 95% *CI*, as well as the corresponding *P* value (http://cran.r-project.org/web/packages/meta/index.html). And then, the original samples were further split into Caucasian, Asian, East Asian and Chinese populations, and the meta-analysis was performed in these subgroups.

### Publication bias analysis and sensitivity analysis

We first evaluated the publication bias of the studies used in dominant, allele and recessive model, respectively, by the two common checking methods, the Begg’s test^67^ and Egger’s test^68^. The threshold of significant publication bias was set as *P* < 0.05. Then, we used the asymmetry of the funnel plots to describ the results of the publication bias analysis. Finally, for sensitivity analyses, we excluded each study in turn from the whole sample to measure the influence of each study.

### Data availability

All the datasets used in this are available from the corresponding author.

## Results

### Study acquisition and data extraction

By a keyword search in the publicly available databases and a screening according to the criteria, a total 46 studies from 36 articles were identified which mainly involved in Caucasian and Asian populations. Moreover, a study about Sweden population was selected from AlzGene database, and three studies involved in Asian populations were identified by the citation check using Google Scholar.

**Figure 1:**
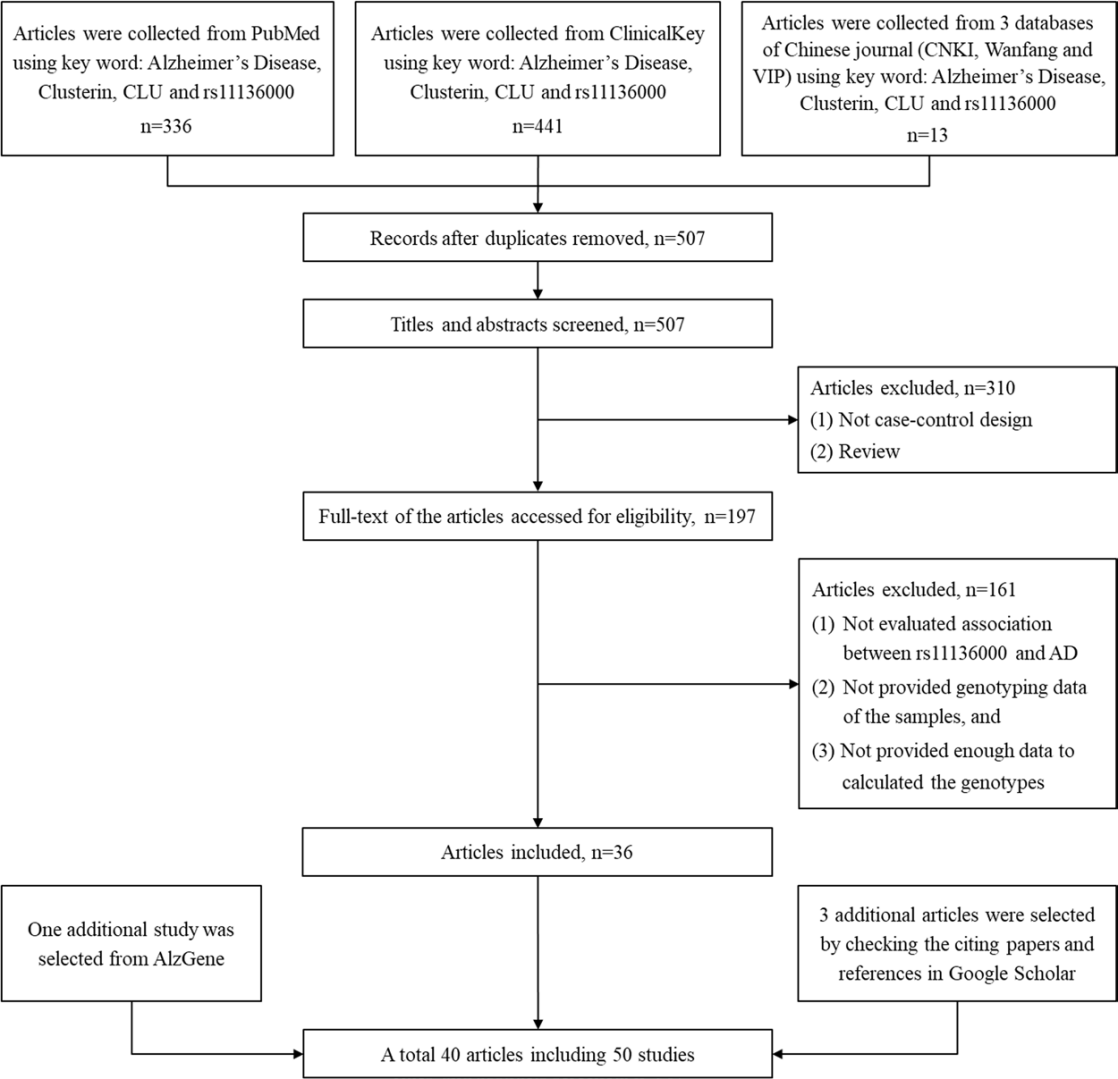
Flow chart of selecting studies for analyzing the association between rs11136000 polymorphism and AD.

Figure 1 showed the workflow of selection. Then, the related data of these 50 studies were extracted, and the main information was described in Table 1 (the detailed genotype data, the *OR* value and its 95% *CI*, as well as the corresponding *P* value were shown in Supplementary Table S1).

### Hardy–Weinberg equilibrium test

We calculated the *P* value of HWE to assess the genotype distribution of rs11136000 polymorphism in AD patients and controls separately. Using a significance level of *P* < 0.01, we observed that a few of the samples deviated from HWE, including the case samples from the study of Yu *et al*. (*P* = 9.0 × 10^−3^) and Gu *et al*. (*P* = 2.0 × 10^−4^), and the control samples from the study of Rezazadeh *et al*. (*P* = 1.0 × 10^−4^), Gu *et al*. (*P* = 1.0 × 10^−4^) and Lin *et al*. (*P* = 9.0 × 10^−3^). More detailed information about the results of the HWE test was described in Supplementary Table S2.

### Heterogeneity Test and Meta-analysis

**Figure 2:**
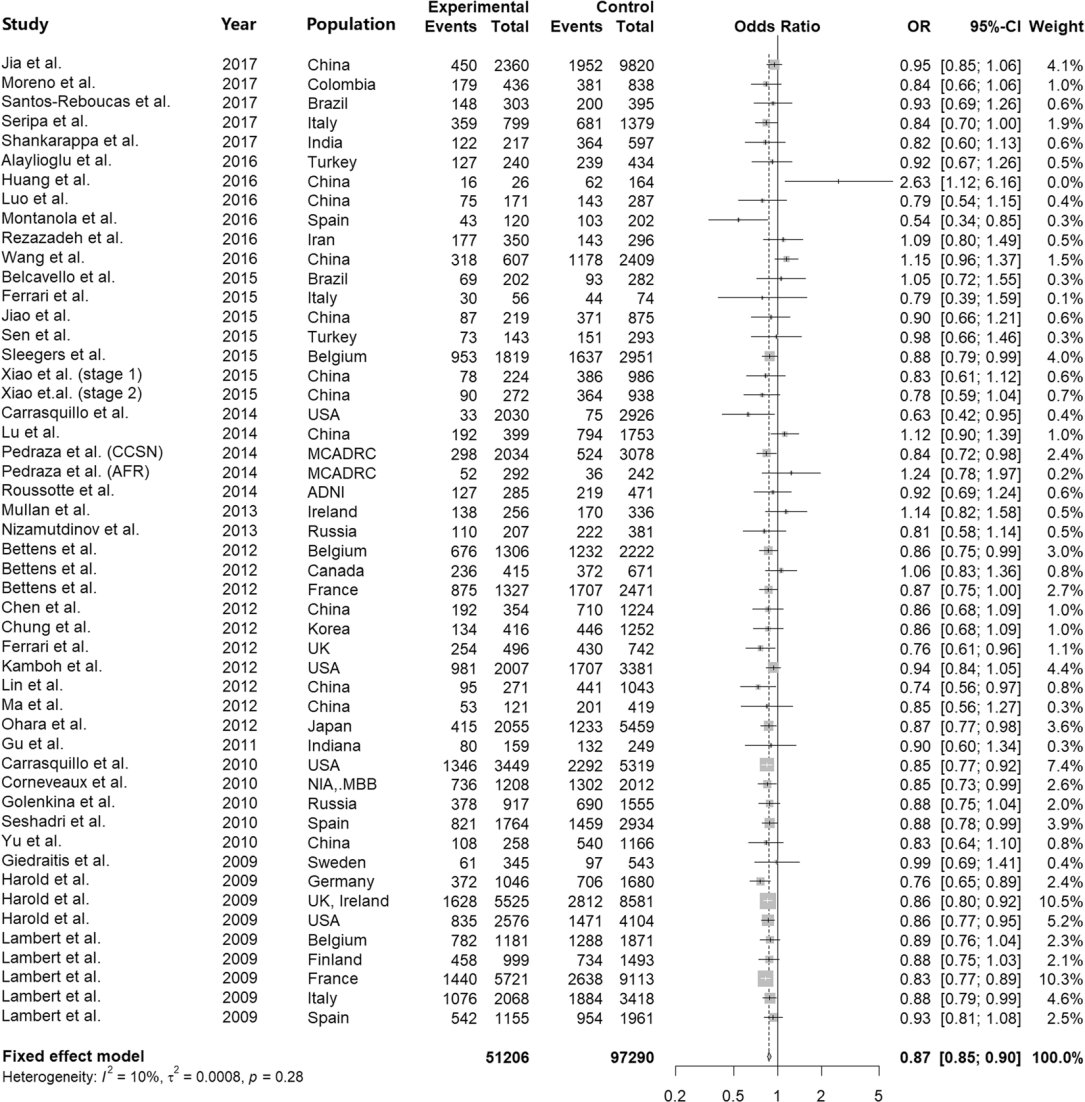
Forest plot for the meta-analysis of rs11136000 polymorphism using allele model. All the 50 selected studies are used to meta-analysis of the allele contrast (T versus C) by the fixed effect model (Mantel-Haenszel) because the genetic heterogeneity is not significant. The minor allele (T) of rs11136000 was significantly associated with a decreased risk of AD.

After the test, we found that there is no the significant genetic heterogeneity of rs11136000 polymorphism among all of the 50 selected studies using the dominant (*I*^2^ = 0% and *P* = 0.60), allele (*I*^2^ = 10% and *P* = 0.28) and recessive model (*I*^2^ = 33% and *P* = 0.04). Therefore, the meta-analysis with fixed effect model was performed to assess the association between rs11136000 and the risk of AD, and we found significant results in all the three models. In particular, the significant association between the minor allele (T) of rs11136000 and a decreased risk of AD was identified in the allele (*OR* = 0.875, 95% *CI* = 0.854–0.896, *P* < 0.0001) (Fig. 2), dominant (*OR* = 0.848, 95% *CI* = 0.817–0.879, *P* < 0.0001) and recessive model (*OR* = 0.822, 95% *CI* = 0.779–0.868, *P* < 0.0001) (Supplementary Figs S1 and S2).

### Subgroup Analysis

**Figure 3:**
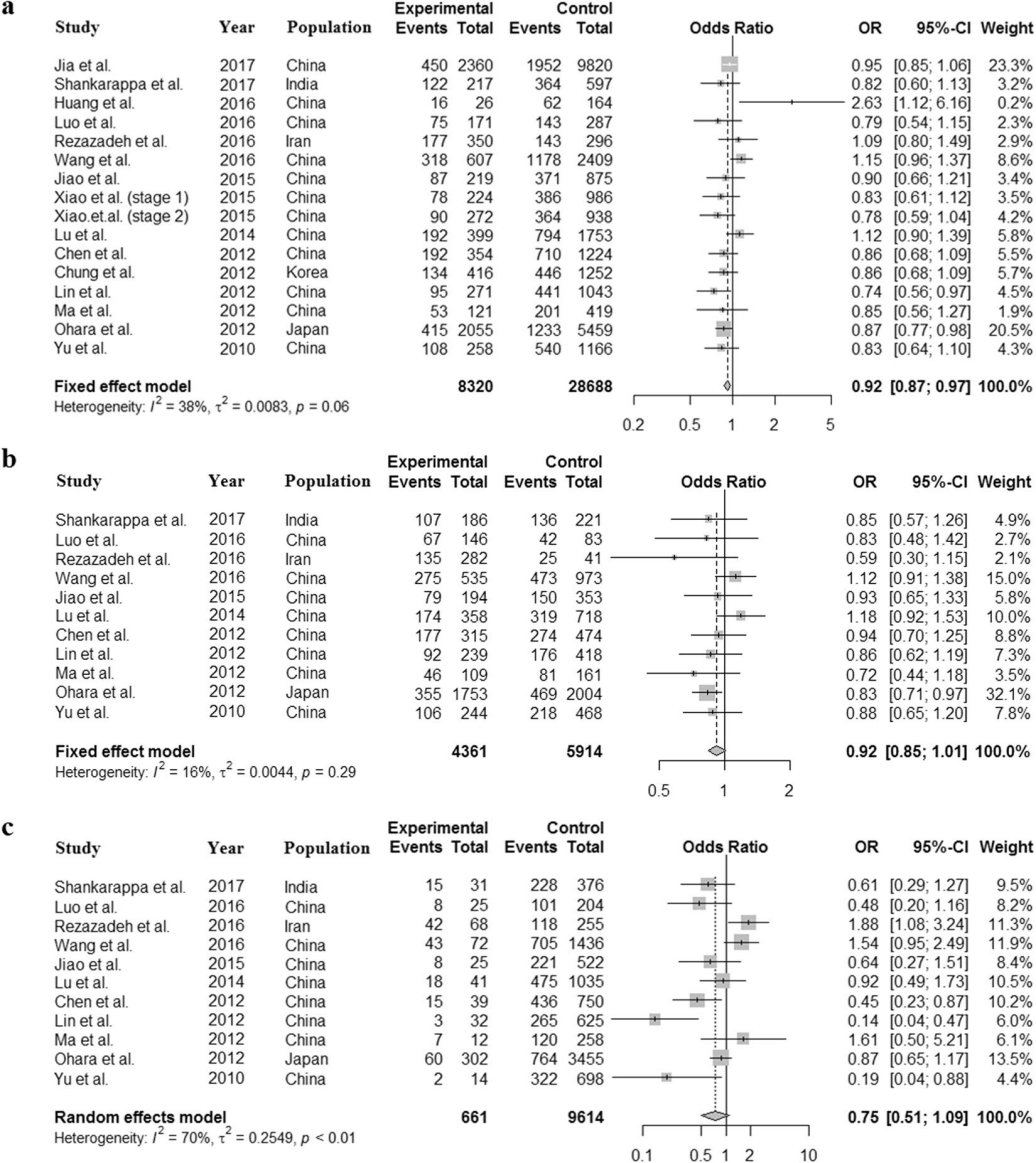
Forest plot for the meta-analysis of rs11136000 polymorphism in Asian population. Only a weak association between rs11136000 polymorphism and AD is observed in the allele model (**a**), but not the dominant (**b**) and recessive model (**c**).

We further performed the meta-analysis in the subgroups to assess the association between rs11136000 and the risk of AD in different ethnicities. Among all the 50 selected studies, the great majority of them involved in Caucasian or Asian ethnicity, except two studies about African and American population, respectively, and four mixed population studies (Table 1). Therefore, we first divided these studies into Caucasian or Asian ethnicity subgroups. We found a significant association between the minor allele (T) of rs11136000 and a decreased risk of AD in Caucasian ethnicity using the allele (*OR* = 0.864, 95% *CI* = 0.842–0.888, *P* < 0.0001), dominant (*OR* = 0.829, 95% *CI* = 0.796–0.864, *P* < 0.0001) and recessive model (*OR* = 0.819, 95% *CI* = 0.774–0.867, *P* < 0.0001) (Supplementary Figs S3–S5). For the Asian ethnicity, however, only a weak association was observed in allele model (*OR* = 0.921, 95% *CI* = 0.871–0.973, *P* = 0.0034) (Fig. 3a), but not the dominant (*OR* = 0.922, 95% *CI* = 0.846–1.005, *P* = 0.0649) (Fig. 3b) and recessive model (*OR* = 0.747, 95% *CI* = 0.511–1.092, *P* = 0.1326) (Fig. 3c).

**Figure 4:**
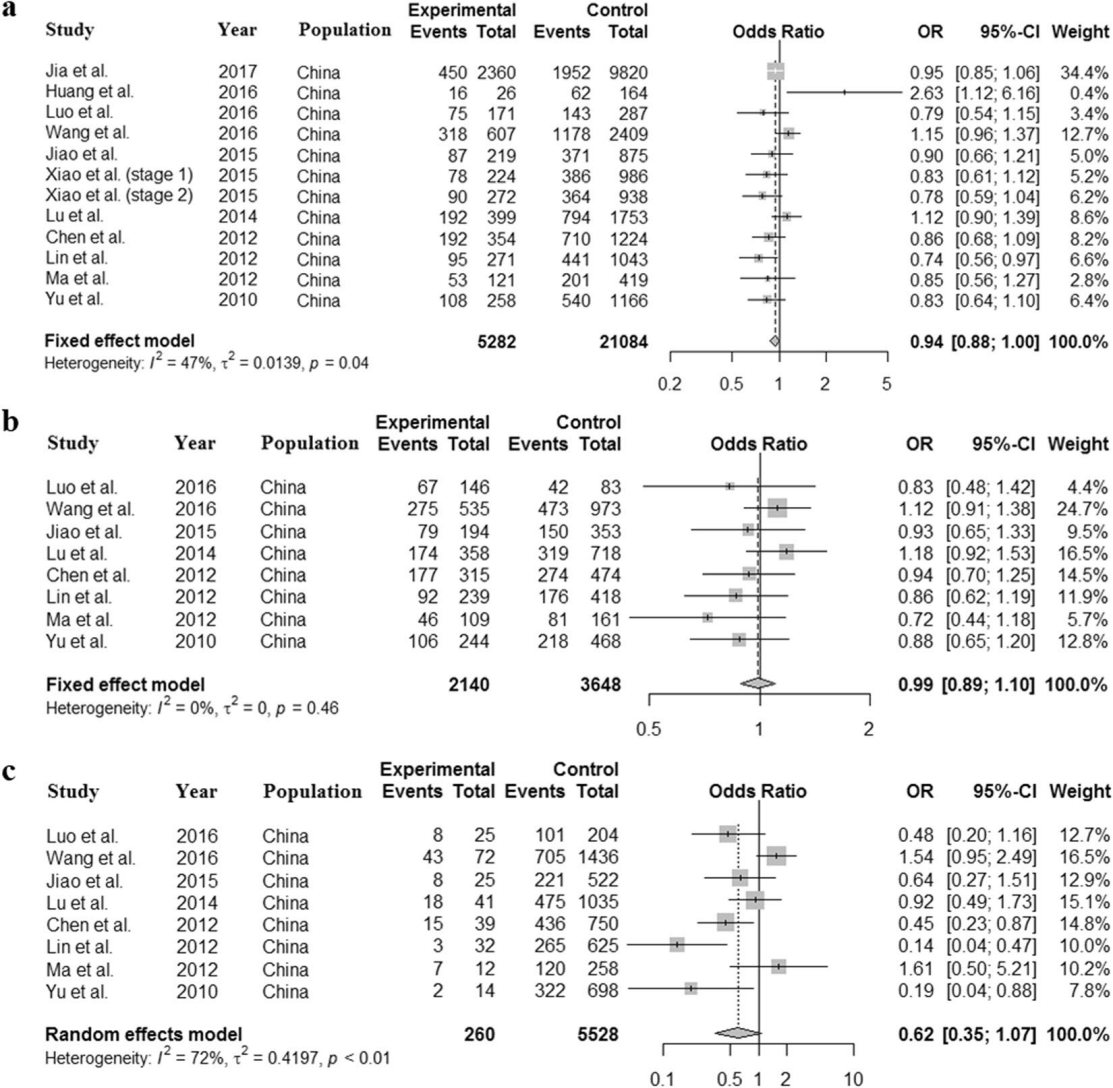
Forest plot for the meta-analysis of rs11136000 polymorphism in Chinese population. The association between rs11136000 polymorphism and AD was not significant in the allele (**a**), dominant (**b**) and recessive model (**c**).

The Asian population in this study was composed of the Indian, Iranian, Korean and Japanese individuals separately from a GWAS study, and the Chinese individuals from 12 GWAS studies. Therefore, we then assessed the association between this SNP and risk of AD in East Asian and Chinese populations. Interestingly, the results of meta-analysis in East Asian population were similar to these in Asian population (Supplementary Figs S6–S8). However, the association was not significant in Chinese population using the allele (*OR* = 0.939, 95% *CI* = 0.878–1.004, *P* = 0.0654) (Fig. 4a), dominant (*OR* = 0.988, 95% *CI* = 0.887–1.101, *P* = 0.8270) (Fig. 4b) and recessive model (*OR* = 0.615, 95% *CI* = 0.355–1.068, *P* = 0.0841) (Fig. 4c), which was different from the findings in the previous studies^29,30^.

Moreover, given that a few samples from four GWAS studies (three Asian populations and a mixed population) deviated from HWE, we further tested whether they affected the accuracy of the results by removing these studies from whole sample, Asian, East Asian and Chinese subgroups, respectively. The results were consistent with what we had been observed previously in whole sample and the subgroups using allele, dominant and recessive model. Table 2 showed the detailed information of the results.

**Table 2:** The results of meta-analysis after removing the studies deviated from HWE.

Ethnicity	Studies	Meta-analysis			Heterogeneity test		Association
		OR	95% IC	P value	I^2^	P value	
***the allele model***							
integrated population	All	0.875	[0.8543; 0.8955]	<0.0001	9.9%	0.2764	significant
integrated population	In HWE	0.875	[0.8524; 0.8960]	<0.0001	11.4%	0.2560	significant
Asian	All	0.927	[0.8777; 0.9786]	0.0034	34.8%	0.0734	significant
Asian	In HWE	0.928	[0.8752; 0.9845]	0.0131	39.4%	0.0706	significant
East Asian	All	0.918	[0.8673; 0.9725]	0.0036	41.8%	0.0501	significant
East Asian	In HWE	0.932	[0.8781; 0.9898]	0.0218	42.8%	0.0573	significant
China	All	0.939	[0.8782; 1.0040]	0.0654	47.1%	0.0355	not significant
China	In HWE	0.962	[0.8959; 1.0332]	0.2884	46.2%	0.0534	not significant
***the dominant model***							
integrated population	All	0.848	[0.8171; 0.8794]	<0.0001	0.0%	0.5996	significant
integrated population	In HWE	0.848	[0.8169; 0.8803]	<0.0001	0.6%	0.4558	significant
Asian	All	0.922	[0.8464; 1.0050]	0.0649	16.0%	0.2917	not significant
Asian	In HWE	0.940	[0.8558; 1.0326]	0.1969	28.1%	0.2037	not significant
East Asian	All	0.934	[0.8545; 1.0205]	0.1304	19.2%	0.2717	not significant
East Asian	In HWE	0.946	[0.8588; 1.0418]	0.2591	36.9%	0.1494	not significant
China	All	0.988	[0.8868; 1.1008]	0.8270	0.0%	0.4601	not significant
China	In HWE	1.026	[0.9072; 1.1612]	0.6794	2.4%	0.4013	not significant
***the recessive model***							
integrated population	All	0.822	[0.7790; 0.8676]	<0.0001	32.6%	0.0387	significant
integrated population	In HWE	0.824	[0.7799; 0.8695]	<0.0001	0.0%	0.5382	significant
Asian	All	0.747	[0.5112; 1.0924]	0.1326	70.5%	0.0002	not significant
Asian	In HWE	0.861	[0.7089; 1.0454]	0.1305	47.7%	0.0631	not significant
East Asian	All	0.675	[0.4441; 1.0254]	0.0654	68.1%	0.0015	not significant
East Asian	In HWE	0.883	[0.7221; 1.0795]	0.2246	51.9%	0.0524	not significant
China	All	0.615	[0.3546; 1.0677]	0.0841	71.8%	0.0008	not significant
China	In HWE	0.892	[0.6767; 1.1750]	0.4154	59.8%	0.0291	not significant

### Publication bias analysis and sensitivity analysis

**Figure 5:**
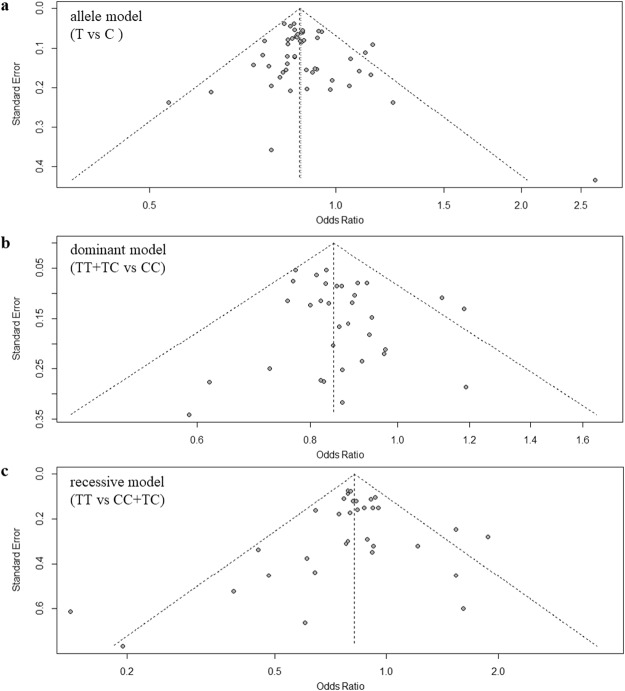
Funnel plot for publication bias analysis of rs11136000 polymorphism in AD using allele, dominant and recessive models.

As the funnel plots show (Fig. 5), we did not identify the significant publication bias in the three genetic models. In particular, the *P* value of Begg’s and Egger’s test is 0.80 and 0.24, respectively, for dominant model. Similarly, the *P* value is 0.43 (Begg’s test) and 0.21 (Egger’s test) for the allele model, and 0.22 (Begg’s test) and 0.61 (Egger’s test) for the recessive model. Moreover, through the sensitivity analysis, for all the three genetic models, we did not found a significant change of the association level between rs11136000 and AD when excluding any of the studies. Supplementary Tables S3–S5 described the related information in detailed.

## Discussion

AD was characterized by accumulation and toxic effect of the Aβ deposits in brain^3^, and previous studies reported that the CLU could markedly influence the fibrillary Aβ formation and accumulation to mediate its toxicity *in vivo*, and likely as one of the most important roles for pathogenesis of AD^6,7^. Then, the subsequent GWAS studies found some variants in CLU were differently distributed between AD patients and controls^11–18^. Among these variants, a significant association was found between the minor allele (T) of rs11136000 and a decreased risk of AD by Harold *et al*.^11^, Lambert *et al*.^12^, Carrasquillo *et al*.^13^ and Seshadri *et al*.^14^. However, these results could not be repeated in other populations by the following studies^19–28^.

Although the two independent meta-analyses found a significant association between the minor allele (T) of rs11136000 and a decreased risk of AD in Caucasian and Asian ethnicities by integrating the data from related GWAS studies published before June 20, 2013 (18 studies) and August 31, 2014 (25 studies), respectively^29,30^, many of the following studies also reported the inconsistent results^31–47^. Moreover, according to our further investigation, the two meta-analyses missed out a total six related GWAS studies published before August 31, 2014^48–52^, and a GWAS study about American and German populations is misclassified to the Asian ethnicity subgroup in Du *et al*.’s meta-analysis^22^. Therefore, we suspected that the small-scale and incompletion or heterogeneity of the samples maybe lead to different results of these studies.

In this study, 50 related GWAS studies (including the 6 missing and 18 novel studies) were selected afresh from seven authoritative sources, and the association level between rs11136000 and risk of AD in Caucasian, Asian and Chinese ethnicity was re-evaluated. We also found a significant association between rs11136000 polymorphism and the decreased risk of AD in Caucasian ethnicity using the dominant (*OR* = 0.829, 95% *CI* = 0.796–0.864, *P* < 0.0001), allele (*OR* = 0.864, 95% *CI* = 0.842−0.888, *P* < 0.0001) and recessive model (*OR* = 0.819, 95% *CI* = 0.774−0.867, *P* < 0.0001). Different from the results of the previous studies, however, rs11136000 polymorphism was found not associated with the risk of AD in Asian ethnicity using the dominant (*OR* = 0.922, 95% *CI* = 0.846–1.005, *P* = 0.0649) and recessive model (*OR* = 0.747, 95% *CI* = 0.511−1.092, *P* = 0.1326), as well as in Chinese population using the dominant (*OR* = 0.988, 95% *CI* = 0.887−1.101, *P* = 0.8270), allele (*OR* = 0.939, 95% *CI* = 0.878–1.004, *P* = 0.0654) and recessive model (*OR* = 0.615, 95% *CI* = 0.355−1.068, *P* = 0.0841).

As far as we know, our meta-analysis about the association of the CLU rs11136000 polymorphism with the risk of AD is by far the largest scale study. The results reveal a significant association between them in Caucasian ethnicity but not Chinese ethnicity, which is consistent with the findings of most of the corresponding GWAS studies. In summary, we believe that these findings can help to improve the understanding of the AD’s pathogenesis.

## Electronic supplementary material

Supporting Information
