# Supplementary material for: Analyzing 74,248 Samples Confirms the Association Between CLU rs11136000 Polymorphism and Alzheimer’s Disease in Caucasian But Not Chinese population
Source: Sci Rep. 2018 Jul 23;8:11062. doi: 10.1038/s41598-018-29450-2 (PMC6056482; doi:10.1038/s41598-018-29450-2)
Supplement: Supplementary file 1 — Supporting Information [file 41598_2018_29450_MOESM1_ESM.docx]

Supporting Information

**CLU rs11136000 Polymorphism increases Alzheimer's disease risk specifically in Caucasian population**

Zhijie Han**^1^**, Jiaojiao Qu**^2^**, Jiehong Zhao**^3^**, Xiao Zou**^2*^**

1 Innovative Drug Research and Bioinformatics Group, School of Pharmaceutical Sciences, Chongqing University, Chongqing, 401331, China

2 Institute of Fungus Resources, College of Life Sciences, Guizhou University, Guiyang, 550025, China.

3 College of Pharmacy, Guiyang University of Chinese Medicine, Guian new area, 550025, China.

* Corresponding Author: Xiao Zou ([xzou@gzu.edu.cn](mailto:xzou@gzu.edu.cn))


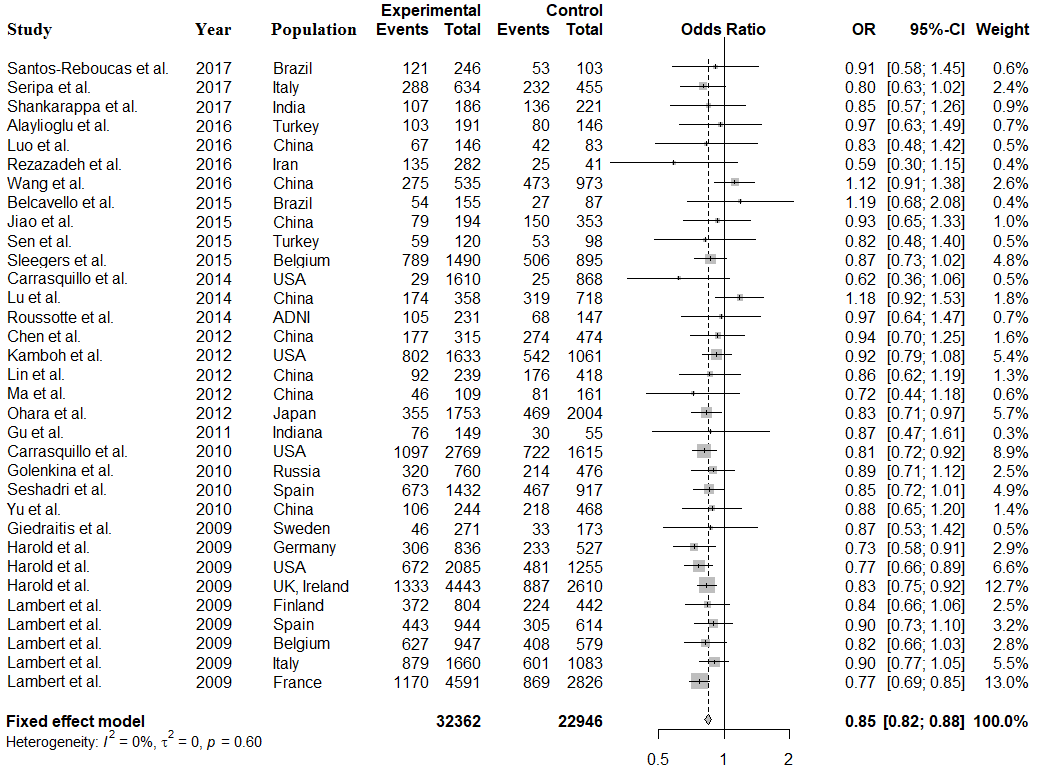


**Figure S1.** The forest plot for the meta-analysis of rs11136000 polymorphism using dominant model.


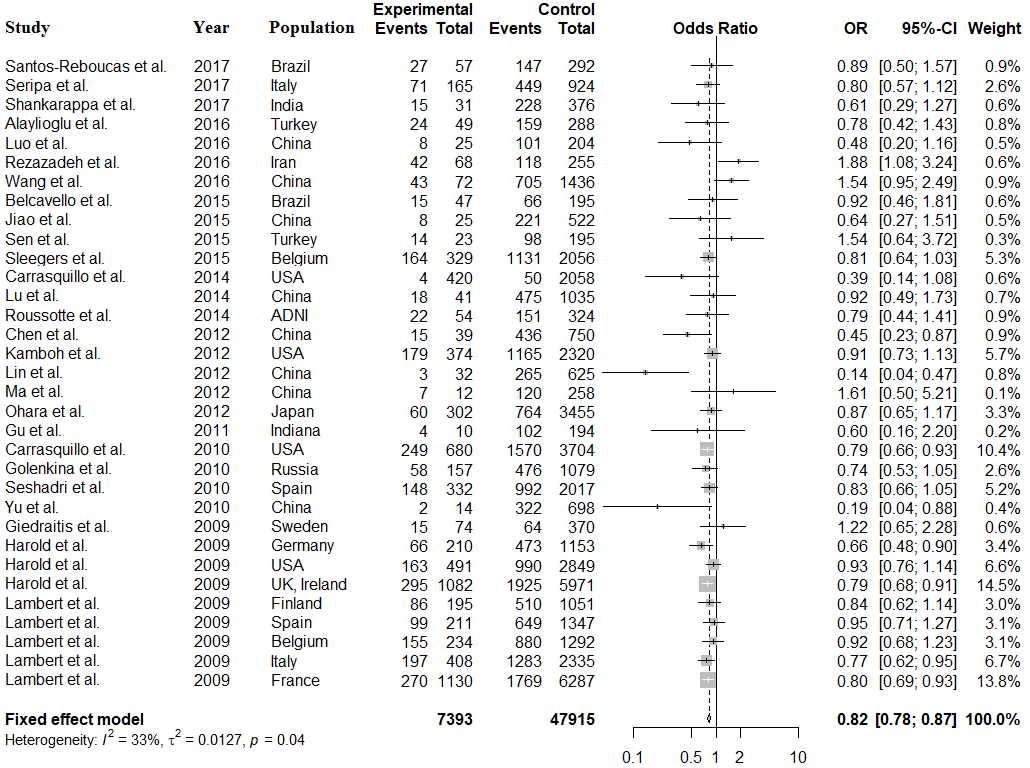


**Figure S2.** The forest plot for the meta-analysis of rs11136000 polymorphism using recessive model.


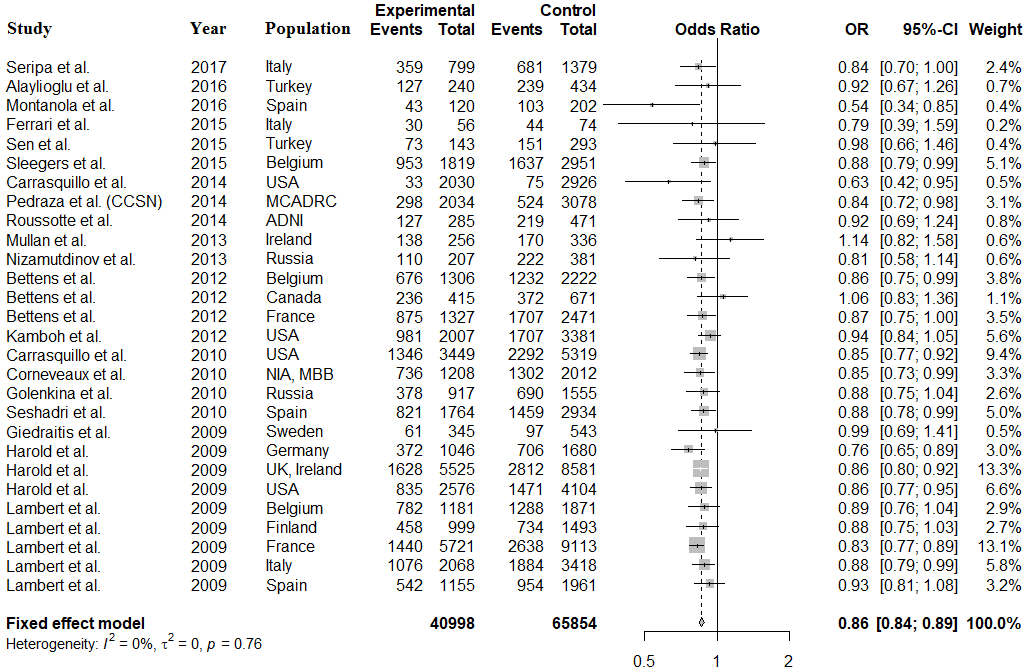


**Figure S3.** The forest plot for the meta-analysis of rs11136000 polymorphism in Caucasian population using allele model.


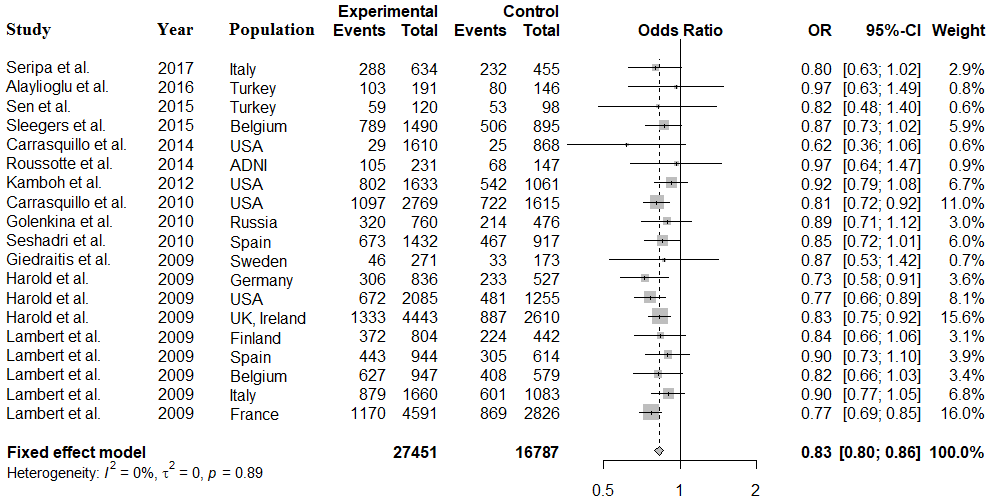


**Figure S4.** The forest plot for the meta-analysis of rs11136000 polymorphism in Caucasian population using dominant model.


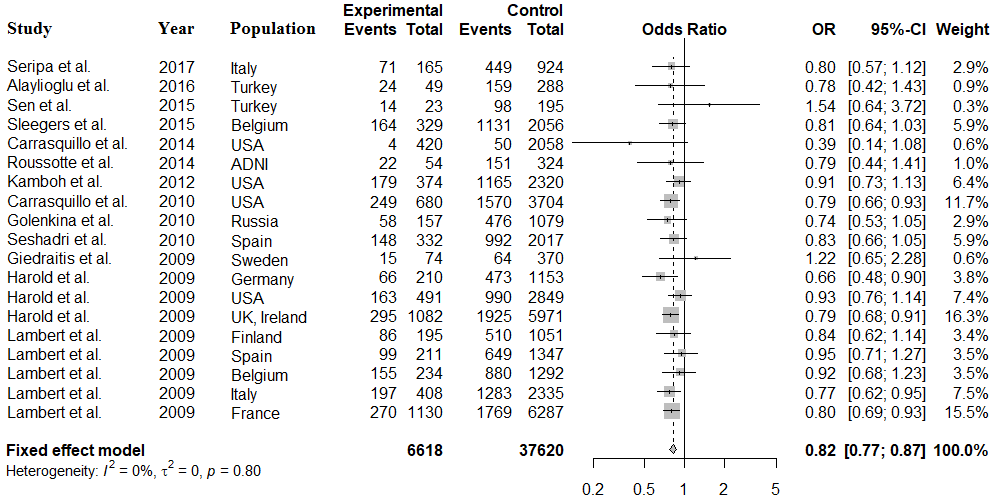


**Figure S5.** The forest plot for the meta-analysis of rs11136000 polymorphism in Caucasian population using recessive model.


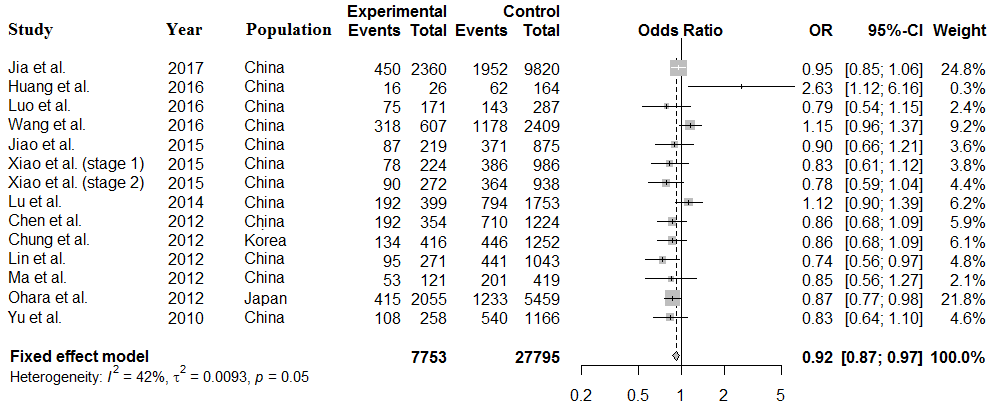


**Figure S6.** The forest plot for the meta-analysis of rs11136000 polymorphism in East Asian population using allele model.


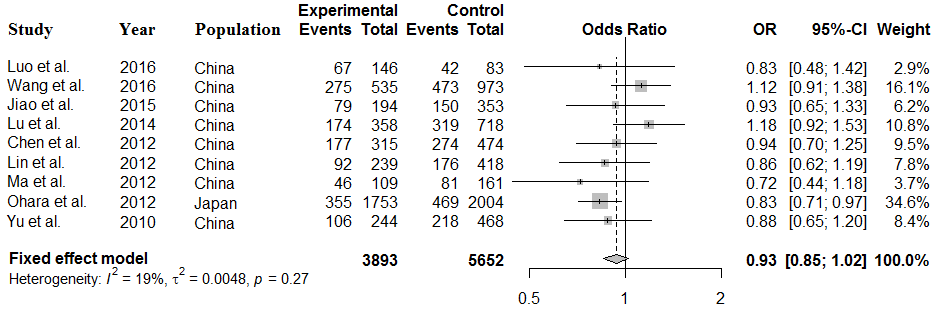


**Figure S7.** The forest plot for the meta-analysis of rs11136000 polymorphism in East Asian population using dominant model.


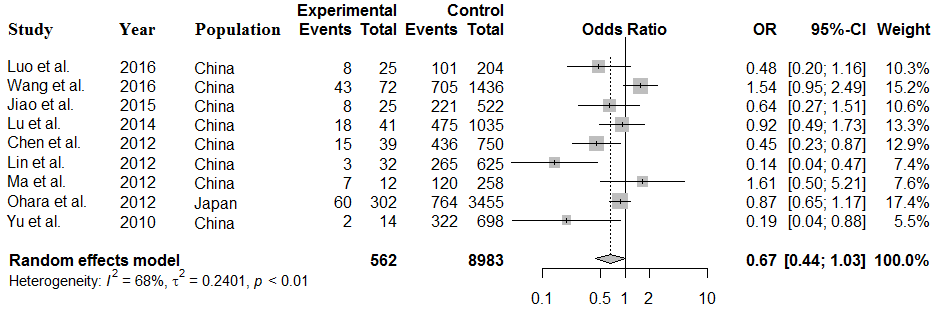


**Figure S8.** The forest plot for the meta-analysis of rs11136000 polymorphism in East Asian population using recessive model.

**Table S1**. the genotype information of the studies included in this meta-analysis

| Study | Year | Country or institution | AD.T | TOTAL.T | AD.C | TOTAL.C | AD.TT | TOTAL.TT | AD.CC | TOTAL.CC | AD.TT+TC | TOTAL.TT+TC | AD.CC | TOTAL.CC | AD.TT | TOTAL.TT | AD.CC+TC | TOTAL.CC+TC |
| --- | --- | --- | --- | --- | --- | --- | --- | --- | --- | --- | --- | --- | --- | --- | --- | --- | --- | --- |
| Shankarappa et al. | 2017 | India | 122 | 217 | 364 | 597 | 15 | 31 | 136 | 221 | 107 | 186 | 136 | 221 | 15 | 31 | 228 | 376 |
| Jia et al. | 2017 | China | 450 | 2360 | 1952 | 9820 | - | - | - | - | - | - | - | - | - | - | - | - |
| Huang et al. | 2016 | China | 16 | 26 | 62 | 164 | - | - | - | - | - | - | - | - | - | - | - | - |
| Wang et al. | 2016 | China | 318 | 607 | 1178 | 2409 | 43 | 72 | 473 | 973 | 275 | 535 | 473 | 973 | 43 | 72 | 705 | 1436 |
| Rezazadeh et al. | 2016 | Iran | 177 | 350 | 143 | 296 | 42 | 68 | 25 | 41 | 135 | 282 | 25 | 41 | 42 | 68 | 118 | 255 |
| Luo et al. | 2016 | China | 75 | 171 | 143 | 287 | 8 | 25 | 42 | 83 | 67 | 146 | 42 | 83 | 8 | 25 | 101 | 204 |
| Jiao et al. | 2015 | China | 87 | 219 | 371 | 875 | 8 | 25 | 150 | 353 | 79 | 194 | 150 | 353 | 8 | 25 | 221 | 522 |
| Xiao et al.(stage 1) | 2015 | China | 78 | 224 | 386 | 986 | - | - | - | - | - | - | - | - | - | - | - | - |
| Xiao et al.(stage 2) | 2015 | China | 90 | 272 | 364 | 938 | - | - | - | - | - | - | - | - | - | - | - | - |
| Lu et al. | 2014 | China | 192 | 399 | 794 | 1753 | 18 | 41 | 319 | 718 | 174 | 358 | 319 | 718 | 18 | 41 | 475 | 1035 |
| Ohara et al. | 2012 | Japan | 415 | 2055 | 1233 | 5459 | 60 | 302 | 469 | 2004 | 355 | 1753 | 469 | 2004 | 60 | 302 | 764 | 3455 |
| Lin et al. | 2012 | China | 95 | 271 | 441 | 1043 | 3 | 32 | 176 | 418 | 92 | 239 | 176 | 418 | 3 | 32 | 265 | 625 |
| Chen et al. | 2012 | China | 192 | 354 | 710 | 1224 | 15 | 39 | 274 | 474 | 177 | 315 | 274 | 474 | 15 | 39 | 436 | 750 |
| Ma et al. | 2012 | China | 53 | 121 | 201 | 419 | 7 | 12 | 81 | 161 | 46 | 109 | 81 | 161 | 7 | 12 | 120 | 258 |
| Chung et al. | 2012 | Korea | 134 | 416 | 446 | 1252 | - | - | - | - | - | - | - | - | - | - | - | - |
| Yu et al. | 2010 | China | 108 | 258 | 540 | 1166 | 2 | 14 | 218 | 468 | 106 | 244 | 218 | 468 | 2 | 14 | 322 | 698 |
| Seripa et al. | 2017 | Italy | 359 | 799 | 681 | 1379 | 71 | 165 | 232 | 455 | 288 | 634 | 232 | 455 | 71 | 165 | 449 | 924 |
| Alaylioglu et al. | 2016 | Turkey | 127 | 240 | 239 | 434 | 24 | 49 | 80 | 146 | 103 | 191 | 80 | 146 | 24 | 49 | 159 | 288 |
| Montanola et al. | 2016 | Spain | 43 | 120 | 103 | 202 | - | - | - | - | - | - | - | - | - | - | - | - |
| Sen et al. | 2015 | Turkey | 73 | 143 | 151 | 293 | 14 | 23 | 53 | 98 | 59 | 120 | 53 | 98 | 14 | 23 | 98 | 195 |
| Sleegers et al. | 2015 | Belgium | 953 | 1819 | 1637 | 2951 | 164 | 329 | 506 | 895 | 789 | 1490 | 506 | 895 | 164 | 329 | 1131 | 2056 |
| Ferrari et al. | 2015 | Italy | 30 | 56 | 44 | 74 | - | - | - | - | - | - | - | - | - | - | - | - |
| Roussotte et al. | 2014 | ADNI | 127 | 285 | 219 | 471 | 22 | 54 | 68 | 147 | 105 | 231 | 68 | 147 | 22 | 54 | 151 | 324 |
| Pedraza et al. | 2014 | MCADRC | 298 | 2034 | 524 | 3078 | - | - | - | - | - | - | - | - | - | - | - | - |
| Carrasquillo et al. | 2014 | USA | 33 | 2030 | 75 | 2926 | 4 | 420 | 25 | 868 | 29 | 1610 | 25 | 868 | 4 | 420 | 50 | 2058 |
| Nizamutdinov et al. | 2013 | Russia | 110 | 207 | 222 | 381 | - | - | - | - | - | - | - | - | - | - | - | - |
| Mullan et al. | 2013 | Ireland | 138 | 256 | 170 | 336 | - | - | - | - | - | - | - | - | - | - | - | - |
| Bettens et al. | 2012 | Belgium | 676 | 1306 | 1232 | 2222 | - | - | - | - | - | - | - | - | - | - | - | - |
| Bettens et al. | 2012 | France | 875 | 1327 | 1707 | 2471 | - | - | - | - | - | - | - | - | - | - | - | - |
| Bettens et al. | 2012 | Canada | 236 | 415 | 372 | 671 | - | - | - | - | - | - | - | - | - | - | - | - |
| Kamboh et al. | 2012 | USA | 981 | 2007 | 1707 | 3381 | 179 | 374 | 542 | 1061 | 802 | 1633 | 542 | 1061 | 179 | 374 | 1165 | 2320 |
| Corneveaux et al. | 2010 | NIA, MBB | 736 | 1208 | 1302 | 2012 | - | - | - | - | - | - | - | - | - | - | - | - |
| Golenkina et al. | 2010 | Russia | 378 | 917 | 690 | 1555 | 58 | 157 | 214 | 476 | 320 | 760 | 214 | 476 | 58 | 157 | 476 | 1079 |
| Seshadri et al. | 2010 | Spain | 821 | 1764 | 1459 | 2934 | 148 | 332 | 467 | 917 | 673 | 1432 | 467 | 917 | 148 | 332 | 992 | 2017 |
| Carrasquillo et al. | 2010 | USA | 1346 | 3449 | 2292 | 5319 | 249 | 680 | 722 | 1615 | 1097 | 2769 | 722 | 1615 | 249 | 680 | 1570 | 3704 |
| Lambert et al. | 2009 | France | 1440 | 5721 | 2638 | 9113 | 270 | 1130 | 869 | 2826 | 1170 | 4591 | 869 | 2826 | 270 | 1130 | 1769 | 6287 |
| Lambert et al. | 2009 | Italy | 1076 | 2068 | 1884 | 3418 | 197 | 408 | 601 | 1083 | 879 | 1660 | 601 | 1083 | 197 | 408 | 1283 | 2335 |
| Lambert et al. | 2009 | Spain | 542 | 1155 | 954 | 1961 | 99 | 211 | 305 | 614 | 443 | 944 | 305 | 614 | 99 | 211 | 649 | 1347 |
| Lambert et al. | 2009 | Belgium | 782 | 1181 | 1288 | 1871 | 155 | 234 | 408 | 579 | 627 | 947 | 408 | 579 | 155 | 234 | 880 | 1292 |
| Lambert et al. | 2009 | Finland | 458 | 999 | 734 | 1493 | 86 | 195 | 224 | 442 | 372 | 804 | 224 | 442 | 86 | 195 | 510 | 1051 |
| Harold et al. | 2009 | USA | 835 | 2576 | 1471 | 4104 | 163 | 491 | 481 | 1255 | 672 | 2085 | 481 | 1255 | 163 | 491 | 990 | 2849 |
| Harold et al. | 2009 | UK,Ireland | 1628 | 5525 | 2812 | 8581 | 295 | 1082 | 887 | 2610 | 1333 | 4443 | 887 | 2610 | 295 | 1082 | 1925 | 5971 |
| Harold et al. | 2009 | Germany | 372 | 1046 | 706 | 1680 | 66 | 210 | 233 | 527 | 306 | 836 | 233 | 527 | 66 | 210 | 473 | 1153 |
| Giedraitis et al. | 2009 | Sweden | 61 | 345 | 97 | 543 | 15 | 74 | 33 | 173 | 46 | 271 | 33 | 173 | 15 | 74 | 64 | 370 |
| Pedraza et al. | 2014 | MCADRC | 52 | 292 | 36 | 242 | - | - | - | - | - | - | - | - | - | - | - | - |
| Belcavello et al. | 2015 | Brazil | 69 | 202 | 93 | 282 | 15 | 47 | 27 | 87 | 54 | 155 | 27 | 87 | 15 | 47 | 66 | 195 |
| Ferrari et al. | 2012 | UK | 254 | 496 | 430 | 742 | - | - | - | - | - | - | - | - | - | - | - | - |
| Gu et al. | 2011 | Indiana | 80 | 159 | 132 | 249 | 4 | 10 | 30 | 55 | 76 | 149 | 30 | 55 | 4 | 10 | 102 | 194 |
| Moreno et al. | 2017 | Colombia | 179 | 436 | 381 | 838 | - | - | - | - | - | - | - | - | - | - | - | - |
| Santos-Rebouças et al. | 2017 | Brazil | 148 | 303 | 200 | 395 | 27 | 57 | 53 | 103 | 121 | 246 | 53 | 103 | 27 | 57 | 147 | 292 |

**Table S2**. The results of the HWE test

| Study | Year | Country or institution | AD.TT | AD.TC | AD.CC | X-squared | P value | CON.TT | CON.TC | CON.CC | X-squared | P value |
| --- | --- | --- | --- | --- | --- | --- | --- | --- | --- | --- | --- | --- |
| Shankarappa et al. | 2017 | India | 15 | 92 | 136 | 0.0114 | 1.0000 | 16 | 63 | 85 | 0.7243 | 0.4456 |
| Luo et al. | 2016 | China | 8 | 59 | 42 | 4.3275 | 0.0320 | 17 | 62 | 41 | 0.7002 | 0.4602 |
| Rezazadeh et al. | 2016 | Iran | 42 | 93 | 25 | 4.9434 | 0.0253 | 26 | 121 | 16 | 39.1800 | 0.0001 |
| Wang et al. | 2016 | China | 43 | 232 | 473 | 4.0405 | 0.0482 | 29 | 231 | 500 | 0.1292 | 0.7258 |
| Jiao et al. | 2015 | China | 8 | 71 | 150 | 0.0128 | 0.8327 | 17 | 98 | 203 | 1.2674 | 0.3137 |
| Lu et al. | 2014 | China | 18 | 156 | 319 | 0.0397 | 0.8863 | 23 | 161 | 399 | 0.4810 | 0.4805 |
| Lin et al. | 2012 | China | 3 | 89 | 176 | 5.1524 | 0.0199 | 29 | 118 | 242 | 6.9362 | 0.0090 |
| Ma et al. | 2012 | China | 7 | 39 | 81 | 0.6245 | 0.4201 | 5 | 58 | 80 | 2.0249 | 0.1732 |
| Chen et al. | 2012 | China | 15 | 162 | 274 | 2.3327 | 0.1586 | 24 | 114 | 200 | 1.8763 | 0.1809 |
| Ohara et al. | 2012 | Japan | 60 | 295 | 469 | 2.0520 | 0.1596 | 242 | 1156 | 1535 | 1.3655 | 0.2500 |
| Yu et al. | 2010 | China | 2 | 104 | 218 | 7.8400 | 0.0090 | 12 | 126 | 250 | 0.6611 | 0.5082 |
| Seripa et al. | 2017 | Italy | 71 | 217 | 232 | 3.0746 | 0.0812 | 94 | 252 | 223 | 2.4967 | 0.1303 |
| Alaylioglu et al. | 2016 | Turkey | 24 | 79 | 80 | 0.4113 | 0.5119 | 25 | 63 | 66 | 2.1955 | 0.1618 |
| Sen et al. | 2015 | Turkey | 14 | 45 | 53 | 0.8197 | 0.3997 | 9 | 52 | 45 | 1.2606 | 0.2732 |
| Sleegers et al. | 2015 | Belgium | 164 | 625 | 506 | 1.8327 | 0.1609 | 165 | 536 | 389 | 0.7860 | 0.3786 |
| Carrasquillo et al. | 2014 | USA | 4 | 25 | 25 | 0.4463 | 0.5207 | 416 | 1165 | 843 | 0.1550 | 0.7058 |
| Roussotte et al. | 2014 | ADNI | 22 | 83 | 68 | 0.1832 | 0.6234 | 32 | 94 | 79 | 0.2105 | 0.6594 |
| Kamboh et al. | 2012 | USA | 179 | 623 | 542 | 1.56E-06 | 1.0000 | 195 | 636 | 519 | 4.80E-05 | 1.0000 |
| Golenkina et al. | 2010 | Russia | 58 | 262 | 214 | 2.8326 | 0.1111 | 99 | 341 | 262 | 0.5069 | 0.4670 |
| Seshadri et al. | 2010 | Spain | 148 | 525 | 467 | 0.0006 | 1.0000 | 184 | 575 | 450 | 0.0002 | 1.0000 |
| Carrasquillo et al. | 2010 | USA | 249 | 848 | 722 | 1.22E-08 | 1.0000 | 431 | 1241 | 893 | 1.91E-05 | 1.0000 |
| Giedraitis et al. | 2009 | Sweden | 15 | 31 | 33 | 2.3430 | 0.1474 | 59 | 166 | 140 | 0.6842 | 0.4504 |
| Harold et al. | 2009 | Germany | 66 | 240 | 233 | 0.1196 | 0.7738 | 144 | 386 | 294 | 0.7917 | 0.3902 |
| Lambert et al. | 2009 | Finland | 86 | 286 | 224 | 0.1185 | 0.7906 | 109 | 323 | 218 | 0.3321 | 0.5711 |
| Lambert et al. | 2009 | Spain | 99 | 344 | 305 | 0.0167 | 0.9357 | 112 | 389 | 309 | 0.3531 | 0.5530 |
| Lambert et al. | 2009 | Belgium | 155 | 472 | 408 | 0.9290 | 0.3524 | 79 | 241 | 171 | 0.1485 | 0.7060 |
| Harold et al. | 2009 | USA | 163 | 509 | 481 | 2.2726 | 0.1389 | 328 | 1085 | 774 | 2.7225 | 0.1018 |
| Lambert et al. | 2009 | Italy | 197 | 682 | 601 | 0.0258 | 0.9116 | 211 | 570 | 482 | 3.6592 | 0.0556 |
| Lambert et al. | 2009 | France | 270 | 900 | 869 | 2.3339 | 0.1294 | 860 | 2561 | 1957 | 0.2104 | 0.6482 |
| Harold et al. | 2009 | UK,Ireland | 295 | 1038 | 887 | 0.1004 | 0.7850 | 787 | 2323 | 1723 | 0.0073 | 0.9313 |
| Belcavello et al. | 2015 | Brazil | 15 | 39 | 27 | 0.0193 | 1.0000 | 32 | 69 | 60 | 2.1710 | 0.1444 |
| Gu et al. | 2011 | Indiana | 4 | 72 | 30 | 21.0340 | 0.0002 | 6 | 67 | 25 | 17.3490 | 0.0001 |
| Santos-Rebouças et al. | 2017 | Brazil | 27 | 94 | 53 | 1.9233 | 0.2099 | 30 | 95 | 50 | 1.7529 | 0.1690 |

**Table S3**. The result of Sensitivity Analysis in allele model

| The omitted study | Year | Country or institution | OR | 95% IC | P value | Tau^2^ | I^2^ |
| --- | --- | --- | --- | --- | --- | --- | --- |
| Jia et al. | 2017 | China | 0.8715 | [0.8507; 0.8927] | < 0.0001 | 0.0007 | 8.30% |
| Moreno et al. | 2017 | Colombia | 0.8751 | [0.8546; 0.8961] | < 0.0001 | 0.0010 | 11.50% |
| Santos-Rebouças et al. | 2017 | Brazil | 0.8743 | [0.8539; 0.8953] | < 0.0001 | 0.0010 | 11.50% |
| Seripa et al. | 2017 | Italy | 0.8754 | [0.8548; 0.8965] | < 0.0001 | 0.0010 | 11.30% |
| Shankarappa et al. | 2017 | India | 0.8750 | [0.8545; 0.8959] | < 0.0001 | 0.0010 | 11.50% |
| Alaylioglu et al. | 2016 | Turkey | 0.8745 | [0.8540; 0.8954] | < 0.0001 | 0.0010 | 11.60% |
| Huang et al. | 2016 | China | 0.8739 | [0.8535; 0.8948] | < 0.0001 | 0.0000 | 0.00% |
| Luo et al. | 2016 | China | 0.8750 | [0.8546; 0.8960] | < 0.0001 | 0.0009 | 11.30% |
| Montanola et al. | 2016 | Spain | 0.8758 | [0.8554; 0.8967] | < 0.0001 | 0.0003 | 4.30% |
| Rezazadeh et al. | 2016 | Iran | 0.8735 | [0.8531; 0.8944] | < 0.0001 | 0.0007 | 8.40% |
| Wang et al. | 2016 | China | 0.8704 | [0.8500; 0.8914] | < 0.0001 | 0.0000 | 0.00% |
| Belcavello et al. | 2015 | Brazil | 0.8741 | [0.8537; 0.8949] | < 0.0001 | 0.0008 | 10.20% |
| Ferrari et al. | 2015 | Italy | 0.8748 | [0.8544; 0.8957] | < 0.0001 | 0.0010 | 11.60% |
| Jiao et al. | 2015 | China | 0.8746 | [0.8541; 0.8955] | < 0.0001 | 0.0010 | 11.70% |
| Sen et al. | 2015 | Turkey | 0.8743 | [0.8539; 0.8952] | < 0.0001 | 0.0009 | 11.30% |
| Sleegers et al. | 2015 | Belgium | 0.8743 | [0.8535; 0.8956] | < 0.0001 | 0.0010 | 11.70% |
| Xiao et al.（stage 1） | 2015 | China | 0.8750 | [0.8545; 0.8959] | < 0.0001 | 0.0010 | 11.60% |
| Xiao et al.（stage 2） | 2015 | China | 0.8754 | [0.8549; 0.8963] | < 0.0001 | 0.0009 | 10.70% |
| Carrasquillo et al. | 2014 | USA | 0.8757 | [0.8553; 0.8966] | < 0.0001 | 0.0006 | 7.60% |
| Lu et al. | 2014 | China | 0.8721 | [0.8517; 0.8931] | < 0.0001 | 0.0002 | 2.80% |
| Pedraza et al. | 2014 | MCADRC | 0.8756 | [0.8550; 0.8968] | < 0.0001 | 0.0009 | 11.20% |
| Pedraza et al. | 2014 | MCADRC | 0.8739 | [0.8535; 0.8948] | < 0.0001 | 0.0006 | 8.10% |
| Roussotte et al. | 2014 | ADNI | 0.8744 | [0.8539; 0.8953] | < 0.0001 | 0.0010 | 11.50% |
| Mullan et al. | 2013 | Ireland | 0.8735 | [0.8530; 0.8943] | < 0.0001 | 0.0006 | 7.40% |
| Nizamutdinov et al. | 2013 | Russia | 0.8750 | [0.8546; 0.8959] | < 0.0001 | 0.0009 | 11.50% |
| Bettens et al. | 2012 | Belgium | 0.8751 | [0.8544; 0.8963] | < 0.0001 | 0.0010 | 11.70% |
| Bettens et al. | 2012 | France | 0.8731 | [0.8527; 0.8941] | < 0.0001 | 0.0006 | 7.80% |
| Bettens et al. | 2012 | Canada | 0.8749 | [0.8542; 0.8961] | < 0.0001 | 0.0010 | 11.70% |
| Chen et al. | 2012 | China | 0.8748 | [0.8544; 0.8958] | < 0.0001 | 0.0010 | 11.70% |
| Chung et al. | 2012 | Korea | 0.8748 | [0.8544; 0.8958] | < 0.0001 | 0.0010 | 11.70% |
| Ferrari et al. | 2012 | UK | 0.8760 | [0.8555; 0.8970] | < 0.0001 | 0.0008 | 9.40% |
| Kamboh et al. | 2012 | USA | 0.8718 | [0.8510; 0.8931] | < 0.0001 | 0.0008 | 9.10% |
| Lin et al. | 2012 | China | 0.8758 | [0.8553; 0.8968] | < 0.0001 | 0.0008 | 9.30% |
| Ma et al. | 2012 | China | 0.8748 | [0.8544; 0.8957] | < 0.0001 | 0.0010 | 11.70% |
| Ohara et al. | 2012 | Japan | 0.8750 | [0.8542; 0.8962] | < 0.0001 | 0.0010 | 11.70% |
| Gu et al. | 2011 | Indiana | 0.8746 | [0.8542; 0.8955] | < 0.0001 | 0.0010 | 11.70% |
| Carrasquillo et al. | 2010 | USA | 0.8770 | [0.8558; 0.8988] | < 0.0001 | 0.0009 | 10.70% |
| Corneveaux et al. | 2010 | NIA, MBB | 0.8753 | [0.8547; 0.8965] | < 0.0001 | 0.0010 | 11.50% |
| Golenkina et al. | 2010 | Russia | 0.8746 | [0.8540; 0.8957] | < 0.0001 | 0.0010 | 11.80% |
| Seshadri et al. | 2010 | Spain | 0.8745 | [0.8537; 0.8957] | < 0.0001 | 0.0010 | 11.70% |
| Yu et al. | 2010 | China | 0.8750 | [0.8545; 0.8959] | < 0.0001 | 0.0010 | 11.60% |
| Giedraitis et al. | 2009 | Sweden | 0.8742 | [0.8538; 0.8951] | < 0.0001 | 0.0009 | 11.00% |
| Harold et al. | 2009 | USA | 0.8774 | [0.8567; 0.8986] | < 0.0001 | 0.0005 | 6.70% |
| Harold et al. | 2009 | UK,Ireland | 0.8767 | [0.8552; 0.8988] | < 0.0001 | 0.0010 | 11.20% |
| Harold et al. | 2009 | Germany | 0.8756 | [0.8546; 0.8970] | < 0.0001 | 0.0010 | 11.50% |
| Lambert et al. | 2009 | France | 0.8744 | [0.8538; 0.8955] | < 0.0001 | 0.0010 | 11.70% |
| Lambert et al. | 2009 | Italy | 0.8747 | [0.8541; 0.8958] | < 0.0001 | 0.0010 | 11.80% |
| Lambert et al. | 2009 | Spain | 0.8803 | [0.8587; 0.9024] | < 0.0001 | 0.0006 | 7.40% |
| Lambert et al. | 2009 | Belgium | 0.8743 | [0.8534; 0.8956] | < 0.0001 | 0.0010 | 11.70% |
| Lambert et al. | 2009 | Finland | 0.8732 | [0.8525; 0.8943] | < 0.0001 | 0.0009 | 10.50% |

**Table S4**. The result of Sensitivity Analysis in dominant model

| The omitted study | Year | Country or institution | OR | 95% IC | P value | Tau^2^ | I^2^ |
| --- | --- | --- | --- | --- | --- | --- | --- |
| Santos-Rebouças et al. | 2017 | Brazil | 0.8473 | [0.8166; 0.8791] | < 0.0001 | 0 | 0.00% |
| Seripa et al. | 2017 | Italy | 0.8489 | [0.8179; 0.8810] | < 0.0001 | 0 | 0.00% |
| Shankarappa et al. | 2017 | India | 0.8477 | [0.8170; 0.8796] | < 0.0001 | 0 | 0.00% |
| Alaylioglu et al. | 2016 | Turkey | 0.8469 | [0.8162; 0.8787] | < 0.0001 | 0 | 0.00% |
| Luo et al. | 2016 | China | 0.8478 | [0.8171; 0.8796] | < 0.0001 | 0 | 0.00% |
| Rezazadeh et al. | 2016 | Iran | 0.8487 | [0.8180; 0.8805] | < 0.0001 | 0 | 0.00% |
| Wang et al. | 2016 | China | 0.8404 | [0.8096; 0.8723] | < 0.0001 | 0 | 0.00% |
| Belcavello et al. | 2015 | Brazil | 0.8465 | [0.8159; 0.8782] | < 0.0001 | 0 | 0.00% |
| Jiao et al. | 2015 | China | 0.8469 | [0.8162; 0.8787] | < 0.0001 | 0 | 0.00% |
| Sen et al. | 2015 | Turkey | 0.8478 | [0.8172; 0.8796] | < 0.0001 | 0 | 0.00% |
| Sleegers et al. | 2015 | Belgium | 0.8468 | [0.8155; 0.8793] | < 0.0001 | 0 | 0.00% |
| Carrasquillo et al. | 2014 | USA | 0.8489 | [0.8182; 0.8807] | < 0.0001 | 0 | 0.00% |
| Lu et al. | 2014 | China | 0.8417 | [0.8110; 0.8735] | < 0.0001 | 0 | 0.00% |
| Roussotte et al. | 2014 | ADNI | 0.8468 | [0.8162; 0.8786] | < 0.0001 | 0 | 0.00% |
| Chen et al. | 2012 | China | 0.8463 | [0.8155; 0.8782] | < 0.0001 | 0 | 0.00% |
| Kamboh et al. | 2012 | USA | 0.8433 | [0.8120; 0.8758] | < 0.0001 | 0 | 0.00% |
| Lin et al. | 2012 | China | 0.8475 | [0.8168; 0.8795] | < 0.0001 | 0 | 0.00% |
| Ma et al. | 2012 | China | 0.8485 | [0.8178; 0.8803] | < 0.0001 | 0 | 0.00% |
| Ohara et al. | 2012 | Japan | 0.8487 | [0.8172; 0.8814] | < 0.0001 | 0 | 0.00% |
| Gu et al. | 2011 | Indiana | 0.8476 | [0.8170; 0.8794] | < 0.0001 | 0 | 0.00% |
| Carrasquillo et al. | 2010 | USA | 0.8513 | [0.8191; 0.8846] | < 0.0001 | 0 | 0.00% |
| Golenkina et al. | 2010 | Russia | 0.8466 | [0.8157; 0.8787] | < 0.0001 | 0 | 0.00% |
| Seshadri et al. | 2010 | Spain | 0.8474 | [0.8160; 0.8799] | < 0.0001 | 0 | 0.00% |
| Yu et al. | 2010 | China | 0.8472 | [0.8165; 0.8792] | < 0.0001 | 0 | 0.00% |
| Giedraitis et al. | 2009 | Sweden | 0.8476 | [0.8169; 0.8794] | < 0.0001 | 0 | 0.00% |
| Harold et al. | 2009 | Germany | 0.8513 | [0.8202; 0.8836] | < 0.0001 | 0 | 0.00% |
| Harold et al. | 2009 | USA | 0.8535 | [0.8217; 0.8865] | < 0.0001 | 0 | 0.00% |
| Harold et al. | 2009 | UK,Ireland | 0.8499 | [0.8171; 0.8840] | < 0.0001 | 0 | 0.00% |
| Lambert et al. | 2009 | Finland | 0.8480 | [0.8170; 0.8801] | < 0.0001 | 0 | 0.00% |
| Lambert et al. | 2009 | Spain | 0.8461 | [0.8151; 0.8783] | < 0.0001 | 0 | 0.00% |
| Lambert et al. | 2009 | Belgium | 0.8485 | [0.8174; 0.8807] | < 0.0001 | 0 | 0.00% |
| Lambert et al. | 2009 | Italy | 0.8445 | [0.8131; 0.8770] | < 0.0001 | 0 | 0.00% |
| Lambert et al. | 2009 | France | 0.8593 | [0.8262; 0.8937] | < 0.0001 | 0 | 0.00% |

**Table S5**. The result of Sensitivity Analysis in recessive model

| The omitted study | Year | Country or institution | OR | 95% IC | P value | Tau^2^ | I^2^ |
| --- | --- | --- | --- | --- | --- | --- | --- |
| Santos-Rebouças et al. | 2017 | Brazil | 0.8215 | [0.7782; 0.8672] | < 0.0001 | 0.0136 | 34.60% |
| Seripa et al. | 2017 | Italy | 0.8227 | [0.7790; 0.8688] | < 0.0001 | 0.0139 | 34.60% |
| Shankarappa et al. | 2017 | India | 0.8234 | [0.7801; 0.8691] | < 0.0001 | 0.0131 | 33.70% |
| Alaylioglu et al. | 2016 | Turkey | 0.8224 | [0.7791; 0.8681] | < 0.0001 | 0.0136 | 34.60% |
| Luo et al. | 2016 | China | 0.8238 | [0.7805; 0.8695] | < 0.0001 | 0.0124 | 32.60% |
| Rezazadeh et al. | 2016 | Iran | 0.8153 | [0.7723; 0.8607] | < 0.0001 | 0.0065 | 20.00% |
| Wang et al. | 2016 | China | 0.8155 | [0.7724; 0.8609] | < 0.0001 | 0.0084 | 24.40% |
| Belcavello et al. | 2015 | Brazil | 0.8215 | [0.7783; 0.8671] | < 0.0001 | 0.0136 | 34.50% |
| Jiao et al. | 2015 | China | 0.8229 | [0.7797; 0.8686] | < 0.0001 | 0.0133 | 34.20% |
| Sen et al. | 2015 | Turkey | 0.8201 | [0.7770; 0.8656] | < 0.0001 | 0.0120 | 31.90% |
| Sleegers et al. | 2015 | Belgium | 0.8226 | [0.7783; 0.8694] | < 0.0001 | 0.0144 | 34.60% |
| Carrasquillo et al. | 2014 | USA | 0.8246 | [0.7812; 0.8703] | < 0.0001 | 0.0118 | 31.60% |
| Lu et al. | 2014 | China | 0.8214 | [0.7782; 0.8670] | < 0.0001 | 0.0136 | 34.50% |
| Roussotte et al. | 2014 | ADNI | 0.8224 | [0.7791; 0.8681] | < 0.0001 | 0.0137 | 34.60% |
| Chen et al. | 2012 | China | 0.8254 | [0.7820; 0.8713] | < 0.0001 | 0.0109 | 29.80% |
| Kamboh et al. | 2012 | USA | 0.8168 | [0.7726; 0.8635] | < 0.0001 | 0.0138 | 33.50% |
| Lin et al. | 2012 | China | 0.8275 | [0.7840; 0.8734] | < 0.0001 | 0.0066 | 20.50% |
| Ma et al. | 2012 | China | 0.8209 | [0.7778; 0.8664] | < 0.0001 | 0.0126 | 32.90% |
| Ohara et al. | 2012 | Japan | 0.8203 | [0.7766; 0.8665] | < 0.0001 | 0.0140 | 34.50% |
| Gu et al. | 2011 | Indiana | 0.8225 | [0.7794; 0.8681] | < 0.0001 | 0.0134 | 34.30% |
| Carrasquillo et al. | 2010 | USA | 0.8264 | [0.7807; 0.8747] | < 0.0001 | 0.0148 | 34.10% |
| Golenkina et al. | 2010 | Russia | 0.8242 | [0.7804; 0.8704] | < 0.0001 | 0.0136 | 34.10% |
| Seshadri et al. | 2010 | Spain | 0.8216 | [0.7773; 0.8683] | < 0.0001 | 0.0144 | 34.70% |
| Yu et al. | 2010 | China | 0.8244 | [0.7811; 0.8701] | < 0.0001 | 0.0106 | 29.40% |
| Giedraitis et al. | 2009 | Sweden | 0.8198 | [0.7767; 0.8653] | < 0.0001 | 0.0124 | 32.60% |
| Harold et al. | 2009 | Germany | 0.8278 | [0.7837; 0.8743] | < 0.0001 | 0.0123 | 31.70% |
| Harold et al. | 2009 | USA | 0.8143 | [0.7700; 0.8611] | < 0.0001 | 0.0134 | 32.60% |
| Harold et al. | 2009 | UK,Ireland | 0.8279 | [0.7812; 0.8774] | < 0.0001 | 0.0153 | 34.00% |
| Lambert et al. | 2009 | Finland | 0.8216 | [0.7779; 0.8678] | < 0.0001 | 0.0140 | 34.70% |
| Lambert et al. | 2009 | Spain | 0.8179 | [0.7743; 0.8640] | < 0.0001 | 0.0133 | 33.40% |
| Lambert et al. | 2009 | Belgium | 0.8190 | [0.7753; 0.8652] | < 0.0001 | 0.0136 | 34.00% |
| Lambert et al. | 2009 | Italy | 0.8261 | [0.7813; 0.8734] | < 0.0001 | 0.0141 | 33.90% |
| Lambert et al. | 2009 | France | 0.8253 | [0.7789; 0.8745] | < 0.0001 | 0.0155 | 34.40% |
